# Molecular mechanism, diagnosis, and treatment of VACTERL association

**DOI:** 10.3389/fped.2025.1609624

**Published:** 2025-07-07

**Authors:** Manluan Sun, Qiyu Zhao, Bingyu Yang, Lili Liu, Caiquan Zhou, Xinbo Yao, Jia Bu, Jiang Bian, Sai Ge, Zhuangyan Zhu, Binyu Liu

**Affiliations:** ^1^ School of Medicine, Shanxi Datong University, Datong, Shanxi, China; ^2^ Shanxi Province Key Laboratory Cultivation Base Jointly Established by the Department and City of Hormone Metabolic Diseases During Perimenopause, Datong, Shanxi, China; ^3^ Obstetrics and Gynecology Department, The Third People's Hospital of Datong, Datong, Shanxi, China; ^4^ Center of Academic Journal, Shanxi Datong University, Datong, Shanxi, China

**Keywords:** VACTERL association, SHH signaling pathways, cilia-associated signaling pathways, clinical manifestations, differential diagnosis

## Abstract

The VACTERL association is a non-random cluster of congenital malformations involving six distinct conditions: vertebral defects (V), anal atresia (A), cardiac defects (C), tracheoesophageal malformation (TE), renal defects (R), and limb anomalies (L), and is diagnosed when a fetus exhibits three or more of these. Its prevalence is approximately 0.47–0.58 per 10,000 live births. This paper examines the effect of disruptions in the Sonic Hedgehog and cilia-associated signaling pathways, genetically related developmental variations, and maternal environmental factors on the development of VACTERL. In the SHH signaling pathway, we focus on the effects of Sonic Hedgehog ligands, GLI transcription factors, and factors influencing GLI activity (RAC1 and ZIC3), as well as downstream targets (FOXF1 and HOXD13) and other genes and proteins involved in the regulation of SHH signaling (FGF8 and LPP), in the pathogenesis of VACTERL. In this context, ZIC3, which was shown to play a major role in VACTERL pathogenesis in large-scale resequencing, and TRAP1, which was associated with VACTERL pathogenesis in whole-exome resequencing, were highlighted. We also examine the cilia-associated signaling pathways, particularly the role of IFT172 and candidate ciliopathy genes. In addition, we describe the influence of TRAP1, COL11A2, SALL4, WBP11, Copy Number Variants, and maternal environmental factors on VACTERL. We also discuss current diagnostic, therapeutic, and prognostic approaches including prenatal and postnatal treatment options. Furthermore, we highlight the advantages of thoracoscopic surgery over traditional open-surgical treatment while discussing the differential diagnosis of VACTERL from other neonatal malformations with similar symptoms, such as Townes-Brocks syndrome, Baller-Gerold syndrome, and CHARGE syndrome.

## 1 Introduction

**Figure 1:**
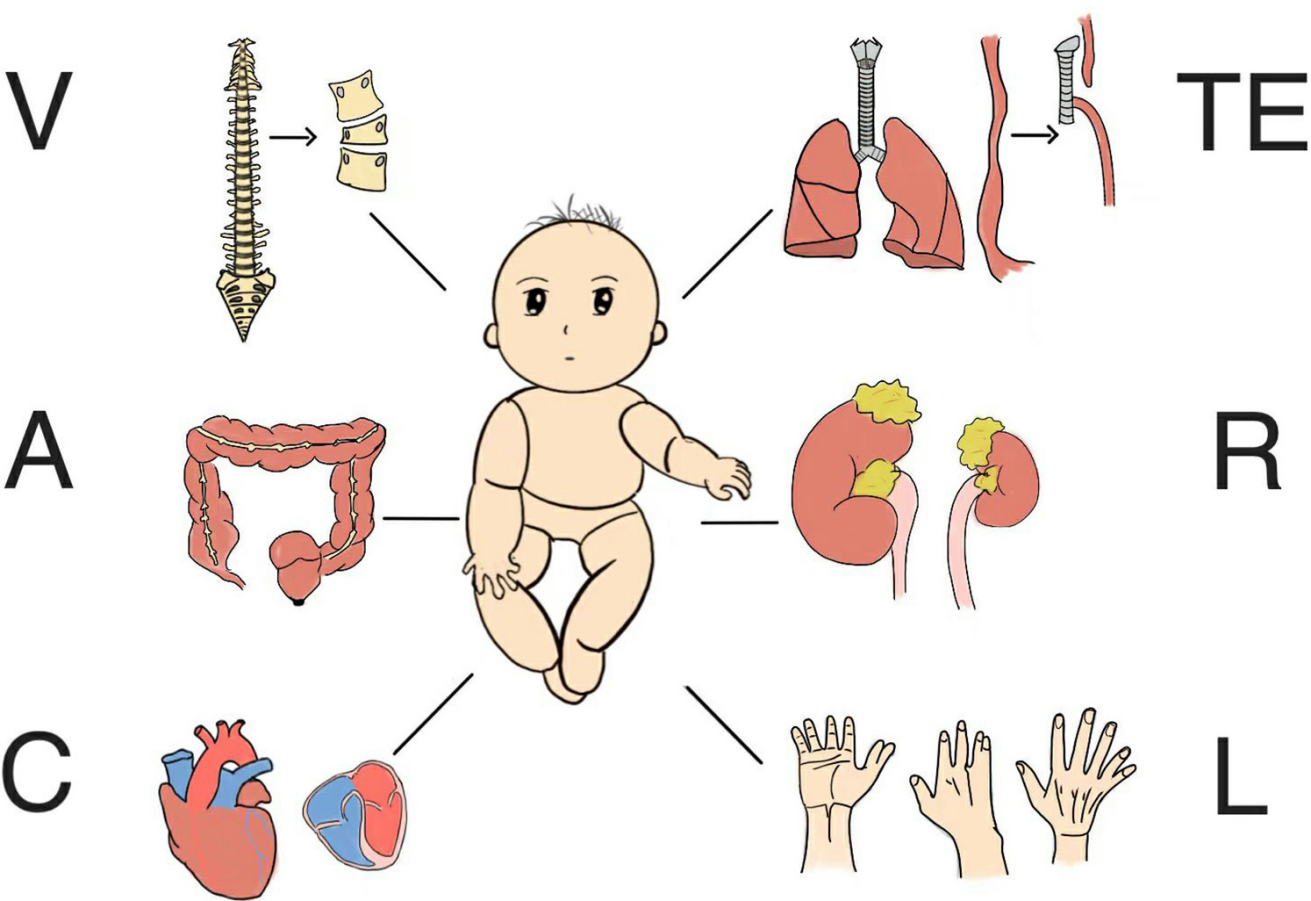
The primary anomalies of VACTERL.

The VACTERL association is a rare, complex congenital malformation with multifactorial causes. According to data published by the European Commission, the prevalence of this condition was 0.47–0.58 per 10,000 live births between 2012 and 2022 (1). This association comprises six primary anomalies (Figure 1): vertebral defects (V), anal atresia (A), cardiac defects (C), tracheoesophageal malformation (TE), renal defects (R), and limb anomalies (L) (2). Quan and Smith first described the disorder as the VATER association in 1972 (3), notably defining R as radial dysplasia, rather than renal defects, as it is understood today. VACTERL usually requires repeated surgeries and may have residual sequelae or even recurrence, and a few patients may not show symptoms or sequelae related to VACTERL until adulthood (4). Thankfully, the severity and likelihood of sequelae have been on the decline in recent years due to medical advances. At the same time, the psychological and social problems caused by VACTERL should not be ignored, including anxiety and depression of patients and their families, and the decline of patients' work efficiency, etc., which require the support and help of all sectors of society (5). A home-centered approach to care seems to be more beneficial for VACTERL patients.

This review discusses the pathogenesis, diagnosis, therapy, and prognosis of VACTERL association. It aims to enrich the diagnostic thinking of clinicians, reduce neonatal sequelae, and enhance neonatal survival by providing ideas for further research into the pathogenesis of VACTERL.

## 2 Clinical manifestation of the VACTERL association

Currently, there is no universally accepted diagnostic standard for the VACTERL association. However, most clinicians and researchers agree that a diagnosis is warranted if a fetus exhibits at least three of the six characteristic congenital abnormalities: vertebral defects, anal atresia, cardiac defects, tracheoesophageal malformation, renal defects, and limb anomalies (6). However, it has not reached full acceptance, with some researchers and clinicians believing that 2 major malformations and one associated feature are sufficient (7). However, some researchers have mentioned that the presence of more than three malformations is not necessarily VACTERL syndrome, and that other neonatal malformations may be present if they are more pronounced and genetically compatible, e.g., a child with concurrent heart defects, anal atresia, vertebral anomalies with early-onset epilepsy, global developmental delay with autistic features, cerebellar hypoplasia, and characteristically dysmorphic facial features (slanted head with downward sloping blepharophimosis, short neck with webbing), the presence of heterozygous *de novo* missense variants of the PACS2 gene should be considered to be on the PACS2 spectrum of disorders (8). An incomplete expression of VACTERL is termed partial VACTERL (pVACTERL) (9).

Vertebral defects are observed in approximately 60%–80% of children with VACTERL. These defects typically include vertebral malformations such as hemivertebrae, butterfly vertebrae, wedge-shaped vertebrae, vertebral fusion, multiple vertebrae anomalies, or absent vertebrae, often accompanied by rib malformations. Rib deformities and spinal defects are also commonly observed. Anal atresia occurs in approximately 55%–90% of cases, presenting with symptoms such as frequent postnatal vomiting, difficulty with nasogastric tube insertion, and absence of stool. Cardiac defects are present in approximately 75% of affected children, most commonly manifesting as congenital heart defects, including atrial septal defect, ventricular septal defect, patent ductus arteriosus, hypoplastic left heart syndrome, transposition of the great arteries, persistent arterial duct, and tetralogy of Fallot (10). Tracheoesophageal fistula with esophageal atresia is found in approximately 50%–80% of VACTERL cases, often manifesting as esophageal atresia or tracheoesophageal fistula, and may be accompanied by pulmonary developmental abnormalities. Clinical symptoms include episodic coughing after swallowing, progressively worsening dysphagia and dyspnea, difficulty inserting nasogastric tubes, and complications such as pneumonia and pleural effusion. Renal defects affect approximately 30%–50% of children with VACTERL and include conditions such as unilateral renal hypoplasia, horseshoe kidney, cystic kidneys, and cystic dysplastic kidneys. Occasionally, ureteral and urogenital tract abnormalities may also be present. Limb anomalies are observed in approximately 40%–70% of cases, including absent or displaced thumbs, polydactyly, syndactyly, and forearm deformities (including radial hypoplasia) (6, 11–14).

These six primary features constitute the core manifestations of VACTERL. Additionally, there are several types of “extension” of VACTERL, which may include abnormalities such as widening of the posterior fossa, hydrocephalus, cerebellar malformations, cerebral hypoplasia, cervical lymphangioma, pulmonary cystadenoma or sequestration, pulmonary hypoplasia, diaphragmatic hernia, and facial asymmetry (hemifacial microsomia). Other associated conditions include absent or hypoplastic nasal bones, cleft lip and/or palate, microtia, external ear malformations, hearing loss, abnormal arteries, moyamoya disease, congenital intestinal malrotation, duodenal stenosis or atresia, single umbilical artery, umbilical cord cyst, umbilical hernia, persistent right umbilical vein, congenital genital abnormalities, cryptorchidism, ambiguous genitalia (14), intrauterine growth restriction (15), pancreatic structural anomalies (16), and biliary tract abnormalities (17). Furthermore, several conditions have been associated with VACTERL, including gray platelet syndrome in neonates (18), Omenn syndrome (19), and spinal muscular atrophy (20).

Based on the statistical analysis of clinical manifestations in affected children (13, 19, 21–38), we derived the probabilities of different malformations: V 60%, A 70%, C 55%, TE 50%, R 65%, L 40%. Two noteworthy phenomena were observed: the incidence of VAR (20% of all cases) was higher than that of other malformation combinations, and the incidence of single umbilical artery and club foot was also higher than that of other malformations.

## 3 Pathogenesis

The pathogenesis of VACTERL remains unclear. Research suggests VACTERL has a multifactorial etiology, involving the interaction of various teratogenic factors. At the molecular level, current research focuses on Sonic Hedgehog (SHH) signaling pathways, cilia-associated signaling pathways, and other genes influencing embryonic development. Maternal gestational status, the fetal environment, and the use of assisted reproductive techniques (ARTs) may also contribute to VACTERL development. These factors influence fetal growth and organogenesis, ultimately contributing to the development of VACTERL.

### 3.1 Sonic hedgehog signaling pathways

SHH signaling pathways may contribute to renal defects within the VACTERL association (39). SHH signaling is involved in dorsoventral axis formation and the development of the foregut, gastrointestinal tract, craniofacial structures, upper and lower limb buds, and the cardiovascular system. In knockout mouse models, the disruption of relevant SHH signaling genes resulted in tracheoesophageal fistula, anal atresia, and ectopic pancreas during foregut and gastrointestinal tract development (40). In limb bud development, ectopic expression of SHH leads to the development of syndactyly (41). In craniofacial development, abnormalities in SHH signaling lead to facial malformations (42).

**Figure 2:**
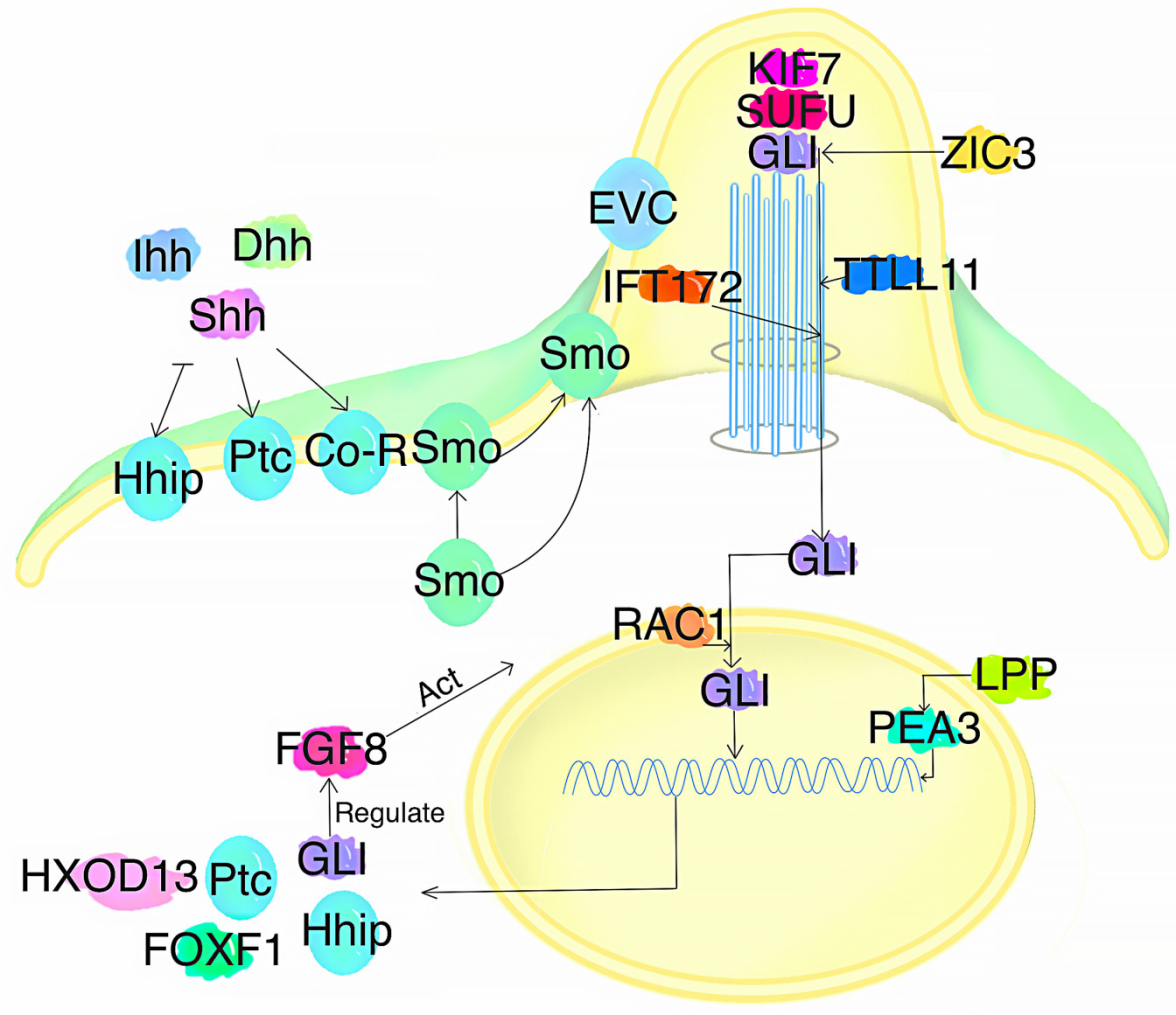
Effect of SHH signaling on the onset of VACTERL. *Shh* ligands activate the SHH signaling pathway. The transcription factors GLI, ZIC3, IFT172, and RAC1 influence signaling, while FOXF1 and HXOD13 act as downstream targets that influence SHH signaling. The FGF8 and LPP (acting through PEA3) modulate SHH signaling. Additionally, the GLI is involved in regulating the FGF8 expression, which affects the SHH pathway. When the above factors are mutated, they may contribute to the pathogenesis of VACTERL.

Hedgehog ligands, transcription factors, downstream targets, and genes involved in the regulation of SHH signaling are involved in the pathogenesis of VACTERL (Figure 2).

#### 3.1.1 Hedgehog ligands

Hedgehog ligands (Shh, Ihh, and Dhh), upon binding to their transmembrane receptor Ptch, activate the signaling regulator SMO, which activates GLI transcription factors (43). Variant in the ligand Shh in SHH signaling prevents normal activation of SHH signaling, leading to malformations. In the study by Kim et al., murine models of *Shh*−/− had a VACTERL phenotype (40), demonstrating that altered Hedgehog ligands lead to the development of VACTERL. However, there are no patients with the SHH variant of VCATERL present and SHH−/− is lethal for humans.

#### 3.1.2 GLI transcription factors

GLI is a transcriptional activator in SHH signaling, responsible for signal transduction from the cytoplasm to the nucleus (43). Variants in *Gli1*, *Gli2*, and *Gli3* within the SHH signaling pathway in mice can potentially cause renal defects (44). Kim et al. demonstrated that *Gli2*−/−, *Gli3*−/−, *Gli2*−/−, and *Gli3*+/− double heterozygous mutant mice develop VACTERL-associated symptoms (40). Jessica Ritter et al. reported on a patient with a GLI1 variant who developed all the symptoms of VACTERL, validating the predictions of this murine models (45).

Beyond the direct effects of GLI, other factors influencing GLI activity may contribute to VACTERL pathogenesis. Rie Seyama et al. reported a case of a suspected VACTERL patient presenting with a RAC1 variant (RAC1-p.Tyr40His). It was shown that the GTP hydrolysis activity of this variant is slightly lower than normal and that RAC1-p.Tyr40His does not activate its effector molecule PAK1 even in the active GTP-bound form, while the downstream effector system may also be hampered by the p.Tyr40His variant, which inactivates the downstream pathway (46). RAC1 activates GLI nuclear translocation in SHH signaling (47), suggesting that RAC1 may influence VACTERL development by affecting GLI nuclear translocation. ZIC3, a member of the GLI superfamily of proteins, plays critical roles in causing left-right pattern defects, midline abnormalities, and cardiac malformations in humans (48–51). It was identified in large-scale resequencing as one of the genes that may contribute to VACTERL (52). In the SHH pathway, it can affect the expression of GLI by interacting with the zinc finger structural domain of GLI, thereby modulating the SHH signaling pathway (53, 54). ZIC3 is also involved in the WNT signaling pathway, transforming growth factor β signaling, and other signaling pathways that regulate the left-right pattern of the embryo (48, 54). Meanwhile, experiments in mice showed that *Zic3* is extremely important for the formation of protozoal embryos and is expressed in the ectoderm, mesoderm, and endoderm of the embryo (55). When *Zic3* is mutated it may affect the development of these germ layers, e.g., abnormal expression of *Zic3* in the ectoderm may lead to neural tube defects, while abnormal expression in the endoderm may lead to defects in the craniofacial region, skeleton, and limbs. Hilger et al. reported four VACTERL patients with ZIC3 variants, three of whom had recurrent disease-causing variant (p.Gly17Cys), and these four patients exhibited ACR, AR, AC, and AR, respectively (52). Notably, in patients with the presence of the ZIC3 p.Gly17Cys variant, the covalent addition of myristoyl esters is disrupted as Gly17 disrupts the covalent addition of myristoyl esters when it substitutes for other small residues, allowing for damage to the N-myristoylation site, which affects the interaction of ZIC3 with other proteins (52). When this site is damaged, the regulatory function of ZIC3 is impaired, which may cause abnormalities in the SHH pathway. Variants in this gene may cause malformations affecting the heart (C), kidneys (R), and limbs (L) (52, 53, 56). Given that the ZIC3 gene is x-linked, variants can result in X-linked VACTERL, and it is also closely related to VACTERL-H.

Furthermore, IFT172, which encodes the intraflagellar transport (IFT) protein (57) essential for GLI function, may also contribute to VACTERL pathogenesis if impaired (58). Jessica Ritter et al. reported on a patient with the IFT172 variant who had all the symptoms of VACTERL (45).

#### 3.1.3 Downstream targets

The forkhead transcription gene FOXF1 is an important downstream target of SHH signaling (59). FOXF1 is expressed in the esophagus, trachea, vertebrae, anus, and reproductive organs. Variants in FOXF1 can lead to malformations in these organs. A study reported a case of a VACTERL patient who presented with a FOXF1 *de novo* variant (p.Gly220Cys) (52). FOXF1 variants have also been shown to cause tracheoesophageal fistula (60). The severity and presentation of each malformation may depend on the residual function and amount of FOX1 protein present (52).

HOXD13 is another downstream target of SHH signaling. Garcia-Barceló et al. reported a case of a VACTERL patient with a HOXD13 variant. Mouse models with the *Hoxd13* variant exhibited limb, intestinal, and genitourinary malformations (61). The mouse model predictions have been confirmed by a number of case reports in which patients with HOXD13 variants also presented with limb anomalies, anal atresia, cardiac defects, and abnormalities of the urinary tract (61). This suggests a possible association between HOXD13 and these malformations.

#### 3.1.4 Genes and proteins regulating the SHH signaling pathway

FGF8 and SHH signaling promote each other through the Hedgehog-FGF signaling axis, which regulates embryonic development. Experiments in mice show that *Fgf8* may act downstream of Hh signaling, while *Gli* also regulates *Fgf8* expression (62). FGF8 activates pathways like Ras—ERK, PI3K—AKT, and phospholipase C gamma-protein kinase C (PLC γ-PKC) to regulate embryonic development (63). Zeidler et al. identified two cases of FGF8 variants in VACTERL patients (64). Given the role of FGF8 in embryonic development, variants may lead to various malformations in the vertebrae (V), anus (A), heart (C), trachea (T), esophagus (E), kidneys (R), and facial regions (63–65). However, since FGF8 is involved in multiple signaling pathways to regulate fetal development, it is possible that a single metabolic pathway is involved. However, it is more likely that multiple pathways are involved in causing neonatal malformations.

Lipoma-preferred partner (LPP) is a LIM domain protein that regulates the function of polyomavirus enhancer activator 3 homolog (PEA3), which is involved in the regulation of SHH signaling as an ETS transcription factor (66). Arrington et al. detected LPP haploinsufficiency in a patient with VACTERL (67). LPP haploinsufficiency may contribute to cardiac anomalies. This variant could disrupt PEA3 function, leading to dysregulated SHH signaling and ultimately abnormal organ development related to VACTERL pathogenesis.

### 3.2 Cilia-associated signaling pathways

In mammalian development, primary cilia are of great significance in the morphogenesis of various organs (45). Faults in the structure and function of primary cilia can lead to a series of developmental abnormalities and metabolic disorders (68, 69). Due to defects in primary cilia, patients may present clinically with malformations similar to those of VACTERL (45, 70–72). Additionally, some studies have found variants in candidate ciliopathy genes in some VACTERL patients, such as TTLL11 (73). Thus, abnormalities in cilia-associated signaling pathways may contribute to VACTERL.

The cilia-associated signaling pathways interact with the SHH signaling pathway. Given that key components of SHH signaling localize to cilia, and SHH-signaling is required for the production of numerous cilia proteins, the structure and function of cilia and SHH signaling are co-dependent (45). Intraflagellar transport (IFT) is one of the structural units of cilia, which is involved in the transport of relevant molecules in the cilia. IFT is a highly conserved bidirectional flow within eukaryotic cilia that transports microtubule proteins and some receptor molecules. Furthermore, IFT is essential in the structural assembly and maintenance of primary cilia. It also plays an important role in cell motility, signaling, embryonic development and organ function (74–77). It stands at the core of the SHH signaling pathway, acting downstream of SMO and upstream of GLI, which is essential for GLI functioning (78). *Avc1* is a hypophenotypic mutant allele of *Ift172*, and *Ift172* encodes a component of IFT (58). Friedland-Little et al. found that a mouse with *Ift172^Avc1^* may develop the VACTERL association with hydrocephalus (VACTERL-H) (58). When IFT172 is mutated, it affects the structural function of IFT, which in turn affects the SHH signaling pathway, IFT, and cytogenesis, leading to the pathogenesis of VACTERL. VACTERL syndrome caused by variants in IFT57 and IFT88 has also been reported (45).In addition to regulating fetal development by affecting the SHH signaling and cilia-associated signaling pathways, the above genes and proteins themselves are involved in fetal development through other pathways. For example, FGF8 can regulate embryonic development through Ras—ERK, PI3K—AKT, and phospholipase C gamma-protein kinase C (PLC γ-PKC).

### 3.3 Other genes involved in VACTERL pathogenesis

In addition to the SHH signaling and cilia-associated signaling pathways mentioned earlier, there are many genes related to VACTERL, such as TRAP1, COLLA2, SALL4, B9D1, FREM1, ZNF157, SP8, ACOT9, and TTLL11.

TNF receptor-associated protein 1 (TRAP1) encoded by TRAP1 is the mitochondrial version of heat shock protein 90 (79), involved in anti-apoptotic and endoplasmic reticulum stress signaling (80). Whole-exome resequencing shows an association between TRAP1 variants and VACTERL pathogenesis (81). In a study of TRAP1 in the Xenopus laevis, it was found that TRAP1 is expressed with developing neural crest cells, somites, renal arches, and pharyngeal arches, and is involved in the developmental processes of several organs. Embryos treated with Gamitrinip-TPP (TRAP1 inhibitor) exhibit abnormalities in craniofacial cartilage, muscle development, and urinary tract development (82).The Xenopus laevis is a model system for studying gene function, so this study suggests a possible mechanism by which human TRAP1 variants lead to VACTERL. In addition, it has been shown that TRAP1 can affect the signaling of the Wnt pathway by regulating the co-receptors of Wnt ligands, LRP5 and LRP6, to modulate embryonic development. When TRAP1 is variant, it leads to down-regulation of LRP5/6 receptors and impaired pathway activation of WNT (83). Saisawat et al. identified TRAP1 as a VACTERL-associated protein and reported two cases of VACTERL patients with TRAP1 variants, presenting as VACTERL, ACTEL and both with missense variants in the HSP90 structural domain of TRAP1 (81). COLLA2 (collagen, type XI, alpha 2) encodes the *α*2 subunit of collagen type XI and is important for bone development and connective tissue formation (84). It has also been reported that COLLA2 may be a candidate gene for vertebral defects and congenital scoliosis (85). Variants in this gene may be involved in the development of vertebral defects (V) in the VACTERL association. SALL4 regulates fetal development by stabilizing embryonic stem cells and is essential for fetal neural tissue, kidney, heart and limb development (86), and according to experimental studies in mice, it can also cooperate with *Gli* to regulate fetal bone development (87, 88). Watanabe et al. suggested that SALL4 haploinsufficiency may lead to VACTERL and identified a patient with SALL4-deficient VACTERL (30). SALL4 variants can adversely affect the anal, heart, kidney, bone, and craniofacial structures (30, 87, 89), leading to the hypothesis that SALL4 variants may be involved in VACTERL pathogenesis. In addition, variants in this gene are involved in the pathogenesis of the thalidomide disaster. Loss-of-function variants in WBP11 which lead to congenital disease in humans may also cause VACTERL. Martin et al. reported four cases of VACTERL due to the WBP11 variant (90). However, Bo Kyung Shin et al. reported a different situation, where WBP11 was mutated but did not produce VACTERL, showing only vertebral anomaly and Sprengel's deformity (91). Additionally, exome sequencing studies have suggested associations between VACTERL association and genes such as FREM1, B9D1, TTLL11, ACOT9, ZNF157, and SP8 (73).

Most of the aforementioned genes, including ZIC3, FOXF1, HOD13, FGF8, LPP, TRAP1, FREM, B9D1, TTLL11, ACOT9, ZNF157, and SP8, are associated with renal defects. This correlation between these genes and clinical symptoms highlights their importance in further elucidating the pathogenesis of VACTERL.

Copy Number Variants (CNV) is also an important cause of VACTERL. The CNV by microdeletion at 19p13.11 patients present with VAR, which has 3 genes (MAP1S, FCHO1, UNC13A/MUNC13A-1) that are mainly associated with autophagy regulation, lattice protein-mediated endocytosis, human T cell development and function, vesicle maturation during synaptic cytokinesis, and maintenance of cellular homeostasis. These genes are involved in cellular processes that are highly active during embryogenesis (92). 1p36.23 duplication (92), 8p23 deletion, 12q23.1 duplication (93), Xq27.1 Microdeletion (94), etc. have also been reported to cause VACTERL.

### 3.4 Other factors related to VACTERL

VACTERL pathogenesis is a complex multifactorial process. Beyond the molecular factors described previously, maternal environmental factors during pregnancy may play a role. These include maternal folate levels before conception and during gestation, pre-existing or gestational diabetes mellitus, chronic lower obstructive pulmonary diseases, and twin pregnancies. The use of ARTs may also be a contributing factor.

Low pre-conception folate levels increase the risk of DNA methylation disturbances, which may contribute to VACTERL development (11). Folate plays a crucial part in one-carbon metabolism, purine and pyrimidine synthesis, and methylation. Maternal folate deficiency leads to decreased levels of levomefolic acid (a folate cycle form) and depletion of S-adenosylmethionine, resulting in decreased DNA cytosine methylation. Research data show that gestational diabetes mellitus increases VACTERL risk (9). Additionally, pregestational diabetes can increase the risk of VACTERL. Statistically, the children of pregnant women with pregestational diabetes have more than three times the risk of developing VACTERL and are prone to cardiac defects and genitourinary malformations, as well as an increased prevalence of craniofacial anomalies, ear anomalies, and hearing loss, compared with the offspring of healthy pregnant women (95). Romy van de Putte et al. showed chronic lower obstructive pulmonary diseases and ART application lead to increased prevalence of VACTERL (95). Carolina I Galarreta et al. also showed that twin pregnancies were significantly associated with the occurrence of microtia in patients with VACTERL (9). However, it has also been suggested that twin pregnancies are not associated with VACTERL development (95).

In addition, environmental factors, such as fetal exposure to estrogen and/or progesterone, statins, and lead *in utero*, may increase the risk of the disease (96). Environmental factors such as drugs, alcohol, food, and nutrition may interfere with gene expression and affect embryonic development, which in turn leads to VACTERL (97). Therefore, understanding these factors is important for taking preventive measures to reduce VACTERL incidence.

Notably, there is an overlap in clinical symptoms between VACTERL and other embryonic developmental malformations, and a common pathogenesis may be present. For example, variants in FGF8 are mainly found in patients with Kallmann syndrome (KS), and VACTERL patients with detectable FGF8 variants have bilateral cryptorchidism, a key phenotype in KS (64). Factors contributing to VACTERL development may also contribute to other diseases. For example, pregestational diabetes is associated with caudal regression syndrome (98), and the *B9d1* and Frem1 genes have been associated with Meckel-Gruber syndrome and the Fraser-related Manitoba oculotrichoanal syndrome (73). The reason for these phenomena may be the presence of the same organ developmental abnormalities in different diseases, and these genes play a vital role in the normal development of the corresponding organs. The association between clinical symptoms and pathogenic factors suggests a possible overlap of pathogenesis between VACTERL and other neonatal malformations, providing ideas for further exploration of the pathogenesis of VACTERL.

Based on the preceding discussion, several key points emerge. First, is there a spectrum of malformations that encompasses VACTERL and neonatal malformations with the same causative genes as its causative genes and similar symptoms? Several congenital disorders demonstrate both genetic overlap and phenotypic similarities with VACTERL. For instance, caudal regression syndrome (associated with CDX2 variants) manifests features overlapping with VACTERL (ARL) as well as its distinctive lower limb deformities (99), while theoculo-auriculo-vertebral spectrum (linked to ZIC3 variants) presents with both VACTERL features and microtia (100). Emerging clinical evidence supports this spectrum hypothesis. In Carolina I Galarreta's study of 263 VACTERL patients, ear anomalies were found in 10.2%, ear malformations were found in 5.9%, hearing loss was found in 13.9%, and orofacial clefts were found in 3.1% (9). Notably, these features—ear malformations, hearing loss, and orofacial clefts—constitute hallmark manifestations of other distinct syndromes such as CHARGE syndrome with Ear malformations and Cleft lip and/or palate, and may be accompanied by VTECRL; Goldenhar syndrome with Ocular and auricular malformations, and may be accompanied by VACRL. This phenotypic overlap raises the possibility that these congenital diseases may exist in the same spectrum of malformations, rather than different ones.

Second, although some studies have pointed out that the above genes are involved in the pathogenesis of VACTERL, large-scale resequencing has shown that many of the above genes do not play a major role in the pathogenesis of VACTERL, such as FGF8 (101), and even, the causative genes confirmed by large-scale resequencing so far are TRAP1 and ZIC3 (101). In addition there is controversy over whether FOXF1 is pathogenic for VACTERL, which was found to be the causative gene in the large-scale resequencing by Alina C Hilger et al (52). However, Corina E Thiem's study refutes this idea (101).Therefore, first, larger-scale sequencing may be required to determine which genes truly cause VACTERL. Second, researchers should not only consider the impact of a single gene but also the interactions between genes and between genes and the environment, studying how these combined effects influence VACTERL. Third, further research is needed on the relationship between this disease and other congenital malformations to better provide genetic counseling for parents and to select more effective prognostic methods.

## 4 Diagnosis and differential diagnosis

The diagnosis of VACTERL is mainly based on the patient's clinical manifestations, as well as relevant imaging studies, but the role of autopsy and molecular diagnosis should not be ignored.

The diagnosis of VACTERL primarily relies on imaging techniques, including x-ray, ultrasound, magnetic resonance imaging (MRI), and radiography. The diagnosis of VACTERL can be divided into prenatal and postnatal periods, with the prenatal period relying mainly on ultrasonography. The following tests are commonly used in the postnatal period, for evaluating cardiac defects and renal defects, ultrasound is the primary diagnostic modality. x-ray is typically used for assessing the spine and limbs. Esophageal atresia and tracheoesophageal fistula can also be detected on x-ray, and radiography is the preferred method for confirming tracheoesophageal malformations (6).

### 4.1 Diagnostic imaging

The radiological diagnosis of VACTERL can be categorized into prenatal and postnatal diagnoses. Prenatal diagnosis primarily relies on ultrasound and MRI, which enable the early detection of abnormalities and make timely intervention possible. Radiologic features that may suggest prenatal VACTERL include colonic dilatation, vertebral defects, amniotic fluid, absence of gastric vesicles, and limb abnormalities (22). However, routine ultrasound screenings can sometimes miss certain conditions. For example, abnormalities in the renal system can be missed due to poor imaging conditions (such as oligohydramnios or even anhydramnios). Small cardiac defects, spinal segmentation anomalies, polydactyly, tracheoesophageal fistula, anal atresia, and genitourinary anomalies are often difficult to detect during prenatal screenings. Also VACTERL-H is not easily detected on ultrasound and most cases of VACTERL-H are detected after birth, but VACTERL-H should be considered when progressive ventricular enlargement is detected (32). Therefore, clinicians should consider the possibility of VACTERL and use a systematic approach to detect common malformations associated with it. This approach involves initially examining the systems with the highest incidence of any anomalies, including the vertebrae (V), heart (C), trachea (T), and esophagus (E), followed by targeted assessments of the anus (A), kidneys (R), and limbs (L) (6). When two or more anomalies are found, it is necessary to examine the remaining four systems for malformations. Postpartum diagnosis primarily involves an x-ray examination of the neonatal vertebrae and limbs. Because spinal deformities typically do not cause discomfort in newborns, scoliosis may not be detected during routine examinations. Furthermore, improper positioning during x-ray imaging can lead to a failure to conduct a thorough examination of the spinal skeletal system, which can result in the missed diagnosis of spinal deformities (102). Moreover, if the clinical presentation includes frequent vomiting, persistent white saliva, or difficulty with gastric tube insertion, esophageal atresia should be suspected; in this case, contrast imaging should be performed for diagnosis and classification, and the possibility of VACTERL association should be considered. Ultrasound should also be used to examine the heart and urinary system for abnormalities, as well as to assess other organ systems for any potential issues. When diagnosing tracheoesophageal fistulas it is important to note that some tracheoesophageal fistulas are not associated with esophageal atresia, in which case fluoroscopic esophagography and bronchoscopy are the mainstay of confirming the diagnosis, as well as CT and endoscopy. False-negative findings on esophagography and CT can be reduced by proper localization and the techniques mentioned above (103).

When diagnosing VACTERL, it is also important to consider the patient's symptoms, such as cough, salivation, cyanosis, feeding difficulties, and respiratory distress, and to consider the possibility of TE and perform the appropriate investigations. It has also been suggested that some TEs without esophageal atresia may not have these symptoms and may present with recurrent pneumonia and respiratory symptoms in late infancy (103), which should be noted by the clinician.

### 4.2 Molecular testing

Due to the phenotypic overlap between VACTERL syndrome and other syndromes and the highly heterogeneous nature of the etiology of VACTERL syndrome, when a differential diagnosis cannot be made on the basis of symptoms and imaging alone, molecular testing may be considered to help differentiate VACTERL syndrome from other syndromes and identify the causative genes, preventing misdiagnosis from affecting the prediction of developmental outcomes and the risk of recurrence. Currently, molecular tests include microarray, karyotype, trio Exome Sequencing, microarray analyses, single-gene testing, specific gene testing, targeted testing, exome sequencing, genome sequencing, and pathway burden test. sequencing, genome sequencing, pathway burden test, and other methods. Prenatally, karyotyping may be considered, which is a useful and relatively inexpensive test for identifying aneuploidies, large, cytogenetically detectable copy number variations, and chromosomal rearrangements that may contribute to the malformations seen in VACTERL associations, in addition to copy number analysis with microarrays, which can also help in the detection of VACTERL; postpartum, a thorough clinical examination is first performed to determine the number and type of congenital malformations, and a comprehensive collection of medical history, family history and imaging data. After the above examination, if the associated disease can be clearly identified, then appropriate molecular testing (Pursue appropriate testing) can be carried out; if the diagnosis can not be clearly identified, microarray analysis can be prioritized, which can reveal potentially pathogenic malformations that can be revealed in a small number of individuals, but a large number of individuals (7).

It is worth noting that molecular genetic analyses aimed at identifying monogenic etiologies may have low diagnostic rates, with monogenic disorders diagnosed in only 5% (5/96) of cases in Jasmina Ćomić's study, which may be due to the fact that VACTERL associations are multifactorial in nature. Exome sequencing is valuable in individuals with atypical features to help identify potential underlying syndromes similar to VACTERL features (104). Exome sequencing is valuable in individuals with atypical features to help identify potential syndromes that resemble VACTERL features (104). However, when a patient has symptoms that closely match those of VACTERL, a better diagnosis can be made based on clinical symptoms and imaging tests. The importance of molecular testing is to prevent misdiagnosis and provide assistance in family counseling.

### 4.3 Fetal autopsy

The importance of fetal autopsy has also attracted our attention. Fetal autopsy helps to correctly diagnose and narrow down the investigation of specific etiologies of congenital anomalies and fetal birth defects through systematic anatomical, histological, and genetic multidimensional analyses, clarifying the combined characteristics of the child's multi-systemic malformations, ruling out confusing disease matches, and compensating for ultrasound's diagnostic limitations (105). It is crucial in confirming prenatal diagnosis, recognizing other malformations, and providing potential etiologies that can direct parental attention to the risk of recurrence (23, 106). It can also identify unexpected congenital anomalies or causes of recurrent miscarriages and stillbirths, helping to search for possible environmental or maternal factors influencing the fetus (107). However, due to cultural, emotional, and other challenges, the use of fetal autopsy is currently low. Fetal autopsies have provided important information for the study of VACTERL syndromes and have promoted further research into the clinical manifestations and pathogenesis of VACTERL syndromes (23).

Although VACTERL is most often diagnosed in infancy, it is worth noting that VACTERL syndrome may also be diagnosed in adulthood (108).

There is currently an issue that deserves our attention: many clinicians have a poor understanding of the range of malformations covered by VACTERL. Some conditions, which do not fall under the category of related malformations, are diagnosed as such. For example, anencephaly and spina bifida are misdiagnosed as V, but they are negatively correlated with VACTERL. Ulnar longitudinal deficiency are rare and their association with VACTERL has not been established, yet they are misdiagnosed as L (26, 109). Some patients with pVACTERL are misdiagnosed as VACTERL (21). These misdiagnoses may lead to a misassessment of the prognosis of patients, so doctors need to deepen their understanding of VACTERL. However, it is also important to recognize that more cases may further expand the scope of VACTERL, which requires further research.

### 4.4 Differential diagnosis

The symptoms of VACTERL are numerous and similar to those of many other diseases; therefore, differential diagnosis is necessary to avoid misdiagnosis.

To better differentiate VACTERL from other diseases, we compared the similarities and differences between VACTERL and other neonatal malformations in terms of major and secondary symptoms, causative genes, and mode of inheritance (Table 1).

**Table 1:** Differential diagnosis.

Disease	Similarity		Difference		Causative gene	Mode of inheritance
	Major symptom	Secondary symptom	Major symptom	Secondary symptom^a^		
Townes-Brocks syndrome (120, 121)	A L	C R	1. Typical thumb malformations without hypoplasia of the radius 2. Dysplastic ears	1. Hearing impairment 2. Foot malformations	*Sall1*	Autosomal dominant
Baller-Gerold syndrome (122)	L	C	1. Coronal craniosynostosis 2. Growth restriction 3. Poikiloderma	1. Intellectual deficiency 2. Imperforate or anterior displacement of the anus 3. Cancer risk	*Recql4*	Autosomal recessive
CHARGE syndrome (123)	TE C R	V L	1. Cranial nerve dysfunction 2. Choanal atresia/stenosis 3. Ocular coloboma 4. Ear malformations 5. Cleft lip and/or palate 6. Endocrine abnormality 7. Developmental delay/intellectual disability 8. Brain anomaly 9. Seizures	1. Gastrointestinal problems 2. Immunodeficiency 3. Neuromuscular problems 4. Dental problems	*Chd7*	Autosomal dominant
Mayer-Rokitansky-Küster-Hauser (MRKH) syndrome (124, 125)	V R	A C L	Genital anomalies (Müllerian duct agenesis, absence of the cranial two-thirds of the vagina, and hypoplasia of the uterus)	1. Hearing impairment 2. Occipital encephalocele 3. Cerebral cysts 4. Cerebellar hypoplasia 5. Seizures 6. Abnormal lobation of the lungs 7. Diaphragmatic agenesis 8. Short stature	Possibly related to *Greb1l*, *Lhx1*, *Hnf1b*, *Tbx6*, and *Wnt9b*	This syndrome occurs sporadically or as an autosomal dominant trait
Goldenhar syndrome (126–128)	V	A C R L	1. Facial asymmetry 2. Ocular and auricular malformations	1. CNS malformations 2. Reproductive system anomalies 3. Respiratory abnormalities	– ^b^	This syndrome occurs sporadically or as an autosomal dominant trait
McKusick-Kaufman syndrome (129)	A C	R	1. Hydrometrocolpos (HMC) in females and genital malformations in males 2. Postaxial polydactyly (PAP)	1. Hirschsprung disease 2. Anteriorly placed anus	*Mkks*	Autosomal recessive
Currarino syndrome (130, 131)	V A^c^	R	Presacral mass	1. Müllerian duct anomalies 2. Developmental delay	*Mnx1*	Autosomal dominant
Holt-Oram syndrome (132)	C L	R	Cardiac conduction disease	1. Craniofacial abnormality 2. Auditory or ocular system abnormalities	*Tbx5*	Autosomal dominant
Caudal regression syndrome (133–135)	VA R L	C	Central nervous system abnormalities	1. Neurogenic bladder 2. Dysmorphic facial 3. Bowel incontinence	Multigenic model *Cdx1* *Cdx2* *Cyp26a1* *Mbtps1* *Plzf* *Sptbn5* *Morn1* *Znf330* *Cltcl1* *Pdzd227* *Vangl1*^d^	Autosomal dominant (*Vangl1)*
Fanconi anemia (136)	R L	V A C TE	1. Short stature 2. Abnormal skin pigmentation 3. Microcephaly 4. Ophthalmic anomalies	1. Endocrine disorders 2. Hearing loss 3. Central nervous system abnormalities 4. Developmental delay and/or intellectual disability 5. Bone marrow failure	At least 23 genes have been identified as being associated with Fanconi anemia	Autosomal recessive, autosomal dominant (RAD51-related FA), and X-linked (FANCB-related FA)

^a^ The major and secondary symptoms here are the primary and secondary symptoms of the diseases listed in the table.

^b^ The causative gene has not been identified, but related chromosomal abnormalities (mosaic and/or partial trisomies) and copy number variations may contribute to the development of the disease.

^c^ The A of Currarino syndrome is similar to but different from VACTERL. Currarino syndrome does not present as anal atresia, and it is an anorectal malformation (usually presenting as chronic constipation).

^d^ The listed genes may be pathogenic for caudal regression syndrome but have not been identified. *Vangl1* has been shown to be the causative gene for the disease in OMIM, which follows autosomal dominant inheritance.

## 5 Treatment and prognosis

VACTERL treatment is broadly categorized into prenatal and postnatal approaches. During the embryonic stage, prenatal diagnosis can identify fetuses with VACTERL, allowing for appropriate genetic counseling and management. Postnatal treatment typically involves surgical correction of specific congenital malformations, with the surgical sequence determined by clinical manifestations, prioritizing life-threatening malformations during the neonatal period (13).

Surgical correction also requires supportive treatments such as infection prevention, nutritional support, and stabilization of the internal environment to ensure the safety of the newborn. In certain organ malformations, thoracoscopic surgery may be used as an alternative to traditional open-surgical treatment. For example, thoracoscopic surgery for congenital esophageal atresia offers advantages such as clear visualization, safety, minimal invasiveness, rapid recovery, and fewer complications, significantly alleviating the symptoms of VACTERL association in affected children (16, 110). Thoracoscopic surgery results in ventilation time, decreased hospitalization, higher anastomotic stenosis, and lower need for long-term tube feeding (111). After esophageal atresia surgery, complications such as anastomotic stricture and tracheoesophageal fistula are common, often requiring secondary corrective surgery (112). Therefore, early surgical intervention is essential. Delaying surgery risks life-threatening complications, such as tracheal obstruction, which can be fatal. It is important to note that when TEF with anal atresia is present, it is important to ensure that the patient is ventilated autonomously, thus avoiding air entry into the atretic gastrointestinal tract in the presence of mechanical ventilation and reducing the risk of aspiration of gastric contents through the fistula (13). Takayuki Masuko mentioned a method for better treatment of persistent cloaca in patients with VACTERL, i.e., intestinal decompression using a continuous transanal drainage system instead of a colostomy without the need for a temporary enterostomy. This avoids the disruption that a colostomy may cause to subsequent procedures such as gastrostomy for esophageal atresia, direct intracardiac surgery requiring an incision near the colostomy, or spinal surgery requiring a prone position postoperatively. Moreover, this approach permits radical repair, which reduces total anesthetic exposure and decreases the risk of surgical site infection, with some improvement in abdominal aesthetics (113). For patients with VACTERL-H, a new treatment option, endoscopic ventriculocystostomy plus Magendie foraminoplasty and plexectomy combined with craniovertebral shunt placement, has recently been proposed by some clinicians, and this approach may improve survival and quality of life in patients with VACTERL-H (25).

Anesthesia in children with VACTERL also requires attention; patients with VACTERL are at elevated risk for anesthesia, such as TE which can complicate airway management and preoperative aspiration, cardiac malformations that can affect hemodynamic stability, renal anomalies that may cause abnormalities in pharmacokinetics and pharmacodynamics, and vertebral malformations that may cause difficulty in surgical positioning. The technique used during anesthesia may be an ultrasound-guided caudal block, which has been shown in some studies to improve the probability of a successful first puncture, and real-time ultrasound monitoring of local anesthetic spread also permits visual confirmation of correct placement. The use of ketamine and dexmedetomidine for sedation and analgesia has proven to be beneficial as it allows for balanced and titratable levels of sedation while maintaining voluntary ventilation. It also provides effective sedation and hemodynamic stability. The risk of respiratory depression and airway complications is minimized by avoiding volatile drugs and opioids (13). Complication rates and mortality in VACTERL patients depend on a variety of factors related to the patient's condition, associated anomalies, surgical technique, and other factors.

Post-surgical care should focus on airway management, gastric tube support, feeding and nutrition management, oral rehabilitation exercises, and maintaining airway patency and assisted ventilation, with emphasis on deoxygenation training.

Patients with VACTERL may continue to have many sequelae after cure (112), some of which are present throughout life or manifest in adulthood (4). V may cause back pain, A is associated with constipation, gastrointestinal obstruction, adhesions, and hemorrhage, C may develop exercise intolerance, TE is associated with dysphagia, poor esophageal motility, asthma, and regurgitation, which may be associated with poor esophageal motility, in addition to choking, tracheal tenderness, and reactive airway disease, R may be associated with kidney stones, pyelonephritis, and recurrent urinary tract infections, and L may present with wrist pain, among others (4, 114). Patients who have had TE may be frequently hospitalized in childhood due to lung infections or stuck food pushes. They take more time than their peers to complete a meal (affecting work-school life) and always have to consider the type of food they eat (115). Also, in addition to the possible negative effects of anesthesia exposure on brain development, frequent hospital admissions can have negative effects such as anxiety, and quality of life (116). Sometimes some patients do not develop other symptoms associated with VACTERL until adulthood. The inconvenience of daily life and physical pain may seriously affect patients' quality of life and mental health. Therefore, comprehensive attention should be given to both the physical and psychological well-being of the patient. In addition to physical therapy, psychological counseling and humane care should be prioritized.

Studies have shown that preschool children aged 5–7 years with VACTERL often experience attention deficits, including concentration difficulties, distractibility, attention-shifting problems, and difficulty sustaining attention, as well as hyperactivity or impulsivity. This may lead to abnormal eating habits and low interest in food (114). These, combined with possible food mass obstruction and intestinal dyskinesia, make feeding patients with VACTERL potentially more complex (115). Some studies have shown no cognitive impairment in patients with VACTERL (114). However, more recent studies have shown that patients with VACTERL are at higher risk for attention deficit hyperactivity disorder (ADHD), autism spectrum disorder (ASD), and intellectual disability (ID) (117). People with VACTERL are also more likely to be depressed (5). This needs to be emphasized by family members, caregivers and followers.

In addition to the physical and mental health of the patient, the mental health of the patient's family should also be emphasized, with parents struggling between the roles of parent and caregiver as they must administer medication, tube feedings, or parenteral feedings to their children (118). Sixty percent of parents of children with TE exhibit fear of choking (115), and parents of children with TE are more likely to be depressed than normal families (5).

Most patients do not like to be treated as patients because of poor health, but would like to be provided with a medical ID in case of emergency (118). Also hospitals where parents can stay with them around the clock, and family-centered care seem to be beneficial for the prognosis of VACTERL patients (118). Long-term multidisciplinary follow-up is essential, and patients and families will also need support for functional and psychosocial changes during adolescence, puberty, and young adulthood (119).

## 6 Summary

The VACTERL association is a rare congenital multiple malformation with an incompletely understood pathogenic mechanism. This review has discussed the clinical manifestations, pathogenesis, differential diagnosis, treatment, and prognosis of VACTERL. Given the diverse presentation of VACTERL and its similarity to many other diseases, it is crucial to strengthen the differential diagnosis to avoid misdiagnosis. The treatment of this condition is symptomatic and should be tailored to individual manifestations. Due to limited understanding and research on VACTERL, current treatment approaches are often insufficient, and the overall level of medical care requires improvement.

## References

[1] European Commission. Prevalence charts and tables. (2022). Available at: https://eu-rd-platform.jrc.ec.europa.eu/eurocat/eurocat-data/prevalence_en (Accessed March 13, 2025).

[2] Temtamy SA Miller JD. Extending the scope of the VATER association: definition of the VATER syndrome. J Pediatr. (1974) 85:345–9. 10.1016/s0022-3476(74)80113-7 4372554

[3] Quan L Smith DW. The VATER association. Vertebral defects, anal atresia, T-E fistula with esophageal atresia, radial and renal dysplasia: a spectrum of associated defects. J Pediatr. (1973) 82:104–7. 10.1016/s0022-3476(73)80024-1 4681850

[4] Raam MS Pineda-Alvarez DE Hadley DW Solomon BD. Long-term outcomes of adults with features of VACTERL association. Eur J Med Genet. (2011) 54:34–41. 10.1016/j.ejmg.2010.09.007 20888933 PMC3033487

[5] Kassa AM Dellenmark-Blom M Thorsell Cederberg J Engvall G Engstrand Lilja H. Children and adolescents with VACTERL association: health-related quality of life and psychological well-being in children and adolescents and their parents. Qual Life Res. (2020) 29:913–24. 10.1007/s11136-019-02364-w 31741214 PMC7142056

[6] Tonni G Koçak Ç Grisolia G Rizzo G Araujo Júnior E Werner H Clinical presentations and diagnostic imaging of VACTERL association. Fetal Pediatr Pathol. (2023) 42:651–74. 10.1080/15513815.2023.2206905 37195727

[7] Solomon BD Bear KA Kimonis V de Klein A Scott DA Shaw-Smith C Clinical geneticists’ views of VACTERL/VATER association. Am J Med Genet A. (2012) 158a:3087–100. 10.1002/ajmg.a.35638 23165726 PMC3507421

[8] Massey H Tennant S Dean J. PACS2, PACS1, and VACTERL: a clinical overlap. Mol Syndromol. (2025) 16:29–32. 10.1159/000539473 39911171 PMC11793877

[9] Galarreta CI Hoyt E Forero L Curry CJ Bird LM. Ear anomalies and hearing loss in patients with VACTERL association and the effect of maternal diabetes. Am J Med Genet A. (2023) 191:2693–702. 10.1002/ajmg.a.63382 37649433

[10] Al-Farqani A Panduranga P Al-Maskari S Thomas E. VACTERL Association with double-chambered left ventricle: a rare occurrence. Ann Pediatr Cardiol. (2013) 6:200–1. 10.4103/0974-2069.115283 24688248 PMC3957460

[11] Lubinsky M. An epigenetic association of malformations, adverse reproductive outcomes, and fetal origins hypothesis related effects. J Assist Reprod Genet. (2018) 35:953–64. 10.1007/s10815-018-1197-2 29855751 PMC6030006

[12] Harjai MM Holla RG Kale R. Full spectrum of VACTERL in new born. Med J Armed Forces India. (2008) 64:84–5. 10.1016/s0377-1237(08)80163-3 27408093 PMC4921771

[13] Costa F Valentim M Ferreira C Santos M. Navigating the anesthetic challenges of vertebral defects, anorectal anomalies, cardiac anomalies, tracheoesophageal Fistula (TEF)/esophageal atresia, renal anomalies, and limb abnormalities (VACTERL) association: a delicate balancing act. Cureus. (2024) 16:e68797. 10.7759/cureus.68797 39376881 PMC11456415

[14] Yuan Z. Clinical analysis and literature review of one case with neonatal VACTERL association Hebei (Master’s thesis). Hebei Medical University, China(Hebei) (2019).

[15] Oral A Caner I Yigiter M Kantarci M Olgun H Ceviz N Clinical characteristics of neonates with VACTERL association. Pediatr Int. (2012) 54:361–4. 10.1111/j.1442-200X.2012.03566.x 22300427

[16] Kimsey KM Barnett GS Keup C Nguyen J Wilsey MJ Smithers CJ Esophageal heterotopic pancreas in an asymptomatic 2-year-old with VACTERL association. JPGN Rep. (2023) 4:e350. 10.1097/pg9.0000000000000350 38034456 PMC10684239

[17] Yoon Y Kim K Yeom SK Lee J Lee Y. A case report of intrahepatic bile duct confluence anomalies in VACTERL syndrome. Medicine (Baltimore). (2018) 97:e12411. 10.1097/md.0000000000012411 30278516 PMC6181584

[18] Alasmari BG Rayees S Althubaiti S Elzubair L Chendeb S. Gray platelet syndrome in a neonate with VACTERL association: a novel homozygous pathogenic variant c.5257C>T in the NBEAL2 gene. Cureus. (2023) 15:e48359. 10.7759/cureus.48359 38060757 PMC10699155

[19] Pangli BK Braddock SR Knutsen AP. Omenn syndrome in a 10-month-old male with athymia and VACTERL association. J Allergy Clin Immunol Glob. (2023) 2:100153. 10.1016/j.jacig.2023.100153 37781660 PMC10509858

[20] Buttle SG McMillan HJ Davila J Bokhaut J Kovesi T Katz SL Respiratory failure in a patient with VACTERL association and concomitant spinal muscular atrophy. Pediatr Pulmonol. (2023) 58:3314–9. 10.1002/ppul.26657 37750602

[21] Delgado J Atkins L Pippin M Jishu J. A case of a newborn presenting with a VACTERL-like association. Cureus. (2024) 16:e75400. 10.7759/cureus.75400 39791075 PMC11717324

[22] Shabnam A Vasugi A Dennis Joseph L Rajan R TM. A precious puzzle: unveiling a suspected VACTERL (vertebral, anal, cardiovascular malformations, tracheo-esophageal Fistula, renal anomalies, and limb defects) association in a 28-day-old neonate. Cureus. (2024) 16:e70143. 10.7759/cureus.70143 39463584 PMC11503426

[23] Jawalkar S Goswami A Patil N Nerune S. VACTERL association in a fetus with a normal genetic profile. Cureus. (2024) 16:e65809. 10.7759/cureus.65809 39219962 PMC11362711

[24] AlBattal NZ Alkhathami AM Alhazmi B Alturki AT BinManie RM. An unusual presentation of pacifier thumb duplication with VACTERL association: case report and review of literature. Int J Surg Case Rep. (2024) 122:110090. 10.1016/j.ijscr.2024.110090 39142182 PMC11379544

[25] Asadov RI Bernard E Enelis B. Endoscopic ventriculocysternostomy, magendie foraminoplasty, and plexusectomy with craniovertebral shunt placement in a pediatric patient with hydrocephalus and VACTERL association: a novel treatment option. Cureus. (2024) 16:e58845. 10.7759/cureus.58845 38784296 PMC11115447

[26] Shimekit MA Yesuf EF Teferi SM Lemma MG. Cartilage within lipomyelomeningocele and ulnar longitudinal deficiency syndrome as VACTERL association, alliance in SHH/GLI3, and Wnt pathway: illustrative case. J Neurosurg Case Lessons. (2024) 7:CASE24177. 10.3171/case24177 38684130 PMC11058404

[27] Acar Z Yılmaz Tuğan B. Congenital superior oblique palsy in a patient with VACTERL association. Saudi J Ophthalmol. (2024) 38:67–70. 10.4103/sjopt.sjopt_297_23 38628410 PMC11017008

[28] Martínez-García J Ordorica-Sandoval SF Rivera-Sainz E Beltrán-Salas MA León-Sicairos N Canizalez-Roman A. Bovine aortic arch with an aberrant left vertebral artery in a 3-year-old boy with VACTERL association: a case report. Am J Case Rep. (2024) 25:e942974. 10.12659/ajcr.942974 38526305 PMC10946695

[29] Le M Wenke K Herrmann J Singer D Lange M. A uncommon case: kasabach-merritt syndrome with VACTERL association. Z Geburtshilfe Neonatol. (2024) 228:298–302. 10.1055/a-2262-8607 38428835

[30] Watanabe D Nakato D Yamada M Suzuki H Takenouchi T Miya F SALL4 Deletion and kidney and cardiac defects associated with VACTERL association. Pediatr Nephrol. (2024) 39:2347–9. 10.1007/s00467-024-06306-8 38329589

[31] Sood A Mishra GV Khandelwal S Suryadevara M Manuja N. Absent thumb and radius in a neonate with tracheo-esophageal Fistula and ventricular septal defect: vACTERL association. Cureus. (2023) 15:e51058. 10.7759/cureus.51058 38269232 PMC10807399

[32] Hong SY Kim SJ Park MH Lee KA. Nonfamilial VACTERL-H syndrome in a dizygotic twin: prenatal ultrasound and postnatal 3D CT findings. Medicina (Kaunas). (2023) 59:1387. 10.3390/medicina59081387 37629676 PMC10456747

[33] Vasta G Tursini S Rovero E Angotti R Molinaro F Briganti V. A case of double cystic esophageal duplication in VACTERL syndrome: the first case report and a review of the literature. Front Pediatr. (2023) 11:1151039. 10.3389/fped.2023.1151039 37152324 PMC10154566

[34] Rao A Gaikwad S Taksande A Wanjari MB. An incidental finding of butterfly vertebrae in a case of vertebral defects, anal atresia, cardiac defects, tracheo-esophageal Fistula, renal anomalies, and limb abnormalities (VACTERL). Cureus. (2023) 15:e33401. 10.7759/cureus.33401 36751248 PMC9899095

[35] Chimenea A García-Díaz L Calderón AM Antiñolo G. Prenatal diagnosis of VACTERL association after early-first trimester SARS-COV-2 infection. Congenit Anom (Kyoto). (2023) 63:44–6. 10.1111/cga.12503 36517451 PMC9877562

[36] Cerron-Vela C Youssef F Cowan KN Davila J. Is horseshoe lung a component of VACTERL spectrum? Case report and review of literature. Radiol Case Rep. (2022) 17:1558–62. 10.1016/j.radcr.2022.02.014 35282320 PMC8914250

[37] Shin BS Kim T Lee HD Ko H Byun JH. Right pulmonary artery originating from ascending aorta (hemitruncus arteriosus) with VACTERL association in a neonate: a case report. Children (Basel). (2022) 9:194. 10.3390/children9020194 35204915 PMC8869914

[38] Pariza PC Stavarache I Dumitru VA Munteanu O Georgescu TA Varlas V VACTERL association in a fetus with multiple congenital malformations—case report. J Med Life. (2021) 14:862–7. 10.25122/jml-2021-0346 35126759 PMC8811671

[39] Reutter H Hilger AC Hildebrandt F Ludwig M. Underlying genetic factors of the VATER/VACTERL association with special emphasis on the “Renal” phenotype. Pediatr Nephrol. (2016) 31:2025–33. 10.1007/s00467-016-3335-3 26857713 PMC5207487

[40] Kim PC Mo R Hui Cc C. Murine models of VACTERL syndrome: role of sonic hedgehog signaling pathway. J Pediatr Surg. (2001) 36:381–4. 10.1053/jpsu.2001.20722 11172440

[41] Shen X Zhang S Zhang X Zhou T Rui Y. Two nonsense GLI3 variants are associated with polydactyly and syndactyly in two families by affecting the sonic hedgehog signaling pathway. Mol Genet Genomic Med. (2022) 10:e1895. 10.1002/mgg3.1895 35218158 PMC9000928

[42] Xu J Iyyanar PPR Lan Y Jiang R. Sonic hedgehog signaling in craniofacial development. Differentiation. (2023) 133:60–76. 10.1016/j.diff.2023.07.002 37481904 PMC10529669

[43] Sigafoos AN Paradise BD Fernandez-Zapico ME. Hedgehog/GLI signaling pathway: transduction, regulation, and implications for disease. Cancers (Basel). (2021) 13:3410. 10.3390/cancers13143410 34298625 PMC8304605

[44] Greenberg D D'Cruz R Lacanlale JL Rowan CJ Rosenblum ND. Hedgehog-GLI mediated control of renal formation and malformation. Front Nephrol. (2023) 3:1176347. 10.3389/fneph.2023.1176347 37675356 PMC10479618

[45] Ritter J Lisec K Klinner M Heinrich M von Schweinitz D Kappler R Genetic disruption of cilia-associated signaling pathways in patients with VACTERL association. Children (Basel). (2023) 10:882. 10.3390/children10050882 37238430 PMC10217539

[46] Seyama R Nishikawa M Uchiyama Y Hamada K Yamamoto Y Takeda M A missense variant at the RAC1-PAK1 binding site of RAC1 inactivates downstream signaling in VACTERL association. Sci Rep. (2023) 13:9789. 10.1038/s41598-023-36381-0 37328543 PMC10275923

[47] Tang C Wu X Ren Q Yao M Xu S Yan Z. Hedgehog signaling is controlled by Rac1 activity. Theranostics. (2022) 12:1303–20. 10.7150/thno.67702 35154488 PMC8771550

[48] Bellchambers HM Barratt KS Diamand KEM Arkell RM. SUMOylation potentiates ZIC protein activity to influence murine neural crest cell specification. Int J Mol Sci. (2021) 22:10437. 10.3390/ijms221910437 34638777 PMC8509024

[49] Cast AE Gao C Amack JD Ware SM. An essential and highly conserved role for Zic3 in left-right patterning, gastrulation and convergent extension morphogenesis. Dev Biol. (2012) 364:22–31. 10.1016/j.ydbio.2012.01.011 22285814 PMC3294024

[50] Bellchambers HM Ware SM. Loss of Zic3 impairs planar cell polarity leading to abnormal left-right signaling, heart defects and neural tube defects. Hum Mol Genet. (2021) 30:2402–15. 10.1093/hmg/ddab195 34274973 PMC8643499

[51] Hossain I Priam P Reynoso SC Sahni S Zhang XX Côté L ZIC2 And ZIC3 promote SWI/SNF recruitment to safeguard progression towards human primed pluripotency. Nat Commun. (2024) 15:8539. 10.1038/s41467-024-52431-1 39358345 PMC11447223

[52] Hilger AC Halbritter J Pennimpede T van der Ven A Sarma G Braun DA Targeted resequencing of 29 candidate genes and mouse expression studies implicate ZIC3 and FOXF1 in human VATER/VACTERL association. Hum Mutat. (2015) 36:1150–4. 10.1002/humu.22859 26294094 PMC4643331

[53] Quinn ME Haaning A Ware SM. Preaxial polydactyly caused by Gli3 haploinsufficiency is rescued by Zic3 loss of function in mice. Hum Mol Genet. (2012) 21:1888–96. 10.1093/hmg/dds002 22234993 PMC3313802

[54] Bellchambers HM Ware SM. ZIC3 In heterotaxy. Adv Exp Med Biol. (2018) 1046:301–27. 10.1007/978-981-10-7311-3_15 29442328 PMC8445495

[55] Elms P Scurry A Davies J Willoughby C Hacker T Bogani D Overlapping and distinct expression domains of Zic2 and Zic3 during mouse gastrulation. Gene Expr Patterns. (2004) 4:505–11. 10.1016/j.modgep.2004.03.003 15261827

[56] Cai R Tan Y Wang M Yu H Wang J Ren Z Detection of novel pathogenic variants in two families with recurrent fetal congenital heart defects. Pharmgenomics Pers Med. (2023) 16:173–81. 10.2147/pgpm.S394120 36923242 PMC10008912

[57] Zheng NX Miao YT Zhang X Huang MZ Jahangir M Luo S Primary cilia-associated protein IFT172 in ciliopathies. Front Cell Dev Biol. (2023) 11:1074880. 10.3389/fcell.2023.1074880 36733456 PMC9887189

[58] Friedland-Little JM Hoffmann AD Ocbina PJ Peterson MA Bosman JD Chen Y A novel murine allele of intraflagellar transport protein 172 causes a syndrome including VACTERL-like features with hydrocephalus. Hum Mol Genet. (2011) 20:3725–37. 10.1093/hmg/ddr241 21653639 PMC3168284

[59] Karolak JA Gambin T Szafranski P Stankiewicz P. Potential interactions between the TBX4-FGF10 and SHH-FOXF1 signaling during human lung development revealed using ChIP-seq. Respir Res. (2021) 22:26. 10.1186/s12931-021-01617-y 33478486 PMC7818749

[60] Gehlen J Giel AS Köllges R Haas SL Zhang R Trcka J First genome-wide association study of esophageal atresia identifies three genetic risk loci at CTNNA3, FOXF1/FOXC2/FOXL1, and HNF1B. HGG Adv. (2022) 3:100093. 10.1016/j.xhgg.2022.100093 35199045 PMC8844277

[61] Garcia-Barceló MM Wong KK Lui VC Yuan ZW So MT Ngan ES Identification of a HOXD13 mutation in a VACTERL patient. Am J Med Genet A. (2008) 146a:3181–5. 10.1002/ajmg.a.32426 19006232

[62] Guzzetta A Koska M Rowton M Sullivan KR Jacobs-Li J Kweon J Hedgehog-FGF signaling axis patterns anterior mesoderm during gastrulation. Proc Natl Acad Sci U S A. (2020) 117:15712–23. 10.1073/pnas.1914167117 32561646 PMC7354932

[63] Al-Qattan MM. The classification of VACTERL association into 3 groups according to the limb defect. Plast Reconstr Surg Glob Open. (2021) 9:e3360. 10.1097/gox.0000000000003360 33680640 PMC7929542

[64] Zeidler C Woelfle J Draaken M Mughal SS Große G Hilger AC Heterozygous FGF8 mutations in patients presenting cryptorchidism and multiple VATER/VACTERL features without limb anomalies. Birth Defects Res A Clin Mol Teratol. (2014) 100:750–9. 10.1002/bdra.23278 25131394

[65] Qi Q Jiang Y Zhou X Lü Y Xiao R Bai J Whole-genome sequencing analysis in fetal structural anomalies: novel phenotype-genotype discoveries. Ultrasound Obstet Gynecol. (2024) 63:664–71. 10.1002/uog.27517 37842862

[66] Guo B Sallis RE Greenall A Petit MM Jansen E Young L The LIM domain protein LPP is a coactivator for the ETS domain transcription factor PEA3. Mol Cell Biol. (2006) 26:4529–38. 10.1128/mcb.01667-05 16738319 PMC1489114

[67] Arrington CB Patel A Bacino CA Bowles NE. Haploinsufficiency of the LIM domain containing preferred translocation partner in lipoma (LPP) gene in patients with tetralogy of fallot and VACTERL association. Am J Med Genet A. (2010) 152a:2919–23. 10.1002/ajmg.a.33718 20949626

[68] Mill P Christensen ST Pedersen LB. Primary cilia as dynamic and diverse signalling hubs in development and disease. Nat Rev Genet. (2023) 24:421–41. 10.1038/s41576-023-00587-9 37072495 PMC7615029

[69] Silva DF Cavadas C. Primary cilia shape hallmarks of health and aging. Trends Mol Med. (2023) 29:567–79. 10.1016/j.molmed.2023.04.001 37137787

[70] Haïm D Roux N Boutaud L Verlin L Quélin C Moncler C Complete loss of IFT27 function leads to a phenotypic spectrum of fetal lethal ciliopathy associated with altered ciliogenesis. Eur J Hum Genet. (2025) 33:387–92. 10.1038/s41431-025-01810-3 39955445 PMC11894207

[71] Li Y Du J Deng S Liu B Jing X Yan Y The molecular mechanisms of cardiac development and related diseases. Signal Transduct Target Ther. (2024) 9:368. 10.1038/s41392-024-02069-8 39715759 PMC11666744

[72] Fitzsimons LA Tasouri E Willaredt MA Stetson D Gojak C Kirsch J Primary cilia are critical for tracheoesophageal septation. Dev Dyn. (2024) 253:312–32. 10.1002/dvdy.660 37776236 PMC10922539

[73] Kolvenbach CM van der Ven AT Kause F Shril S Scala M Connaughton DM Exome survey of individuals affected by VATER/VACTERL with renal phenotypes identifies phenocopies and novel candidate genes. Am J Med Genet A. (2021) 185:3784–92. 10.1002/ajmg.a.62447 34338422 PMC8595524

[74] Huang Y Dong X Sun SY Lim TK Lin Q He CY. ARL3 GTPases facilitate ODA16 unloading from IFT in motile cilia. Sci Adv. (2024) 10:eadq2950. 10.1126/sciadv.adq2950 39231220 PMC11373600

[75] Ku PI Sreeja JS Chadha A Williams DS Engelke MF Subramanian R. Collaborative role of two distinct cilium-specific cytoskeletal systems in driving hedgehog-responsive transcription factor trafficking. Sci Adv. (2025) 11:eadt5439. 10.1126/sciadv.adt5439 40073114 PMC11900865

[76] Pruski M Hu L Yang C Wang Y Zhang JB Zhang L Roles for IFT172 and primary cilia in cell migration, cell division, and neocortex development. Front Cell Dev Biol. (2019) 7:287. 10.3389/fcell.2019.00287 31850339 PMC6890611

[77] Klena N Pigino G. Structural biology of cilia and intraflagellar transport. Annu Rev Cell Dev Biol. (2022) 38:103–23. 10.1146/annurev-cellbio-120219-034238 35767872

[78] May SR Ashique AM Karlen M Wang B Shen Y Zarbalis K Loss of the retrograde motor for IFT disrupts localization of Smo to cilia and prevents the expression of both activator and repressor functions of Gli. Dev Biol. (2005) 287:378–89. 10.1016/j.ydbio.2005.08.050 16229832

[79] Dernovšek J Tomašič T. Following the design path of isoform-selective Hsp90 inhibitors: small differences, great opportunities. Pharmacol Ther. (2023) 245:108396. 10.1016/j.pharmthera.2023.108396 37001734

[80] Montesano Gesualdi N Chirico G Pirozzi G Costantino E Landriscina M Esposito F. Tumor necrosis factor-associated protein 1 (TRAP-1) protects cells from oxidative stress and apoptosis. Stress. (2007) 10:342–50. 10.1080/10253890701314863 17853063

[81] Saisawat P Kohl S Hilger AC Hwang DY Yung Gee H Dworschak GC Whole-exome resequencing reveals recessive mutations in TRAP1 in individuals with CAKUT and VACTERL association. Kidney Int. (2014) 85:1310–7. 10.1038/ki.2013.417 24152966 PMC3997628

[82] Kim HE Kwon T Sim HJ Park TJ. TRAP1 Functions in the morphogenesis of the embryonic kidney. Anim Cells Syst (Seoul). (2025) 29:9–18. 10.1080/19768354.2025.2477789 40098710 PMC11912273

[83] Lettini G Condelli V Pietrafesa M Crispo F Zoppoli P Maddalena F TRAP1 Regulates Wnt/*β*-catenin pathway through LRP5/6 receptors expression modulation. Int J Mol Sci. (2020) 21:7526. 10.3390/ijms21207526 33065966 PMC7589514

[84] Xu R Jiang X Lu J Wang K Sun Y Zhang Y. Genetic variant of COL11A2 gene is functionally associated with developmental dysplasia of the hip in Chinese Han population. Aging (Albany NY). (2020) 12:7694–703. 10.18632/aging.103040 32396528 PMC7244083

[85] Rebello D Wohler E Erfani V Li G Aguilera AN Santiago-Cornier A COL11A2 As a candidate gene for vertebral malformations and congenital scoliosis. Hum Mol Genet. (2023) 32:2913–28. 10.1093/hmg/ddad117 37462524 PMC10508038

[86] Kodytková A Amaratunga SA Zemková D Maratová K Dušátková P Plachý L SALL4 Phenotype in four generations of one family: an interplay of the upper limb, kidneys, and the pituitary. Horm Res Paediatr. (2024) 97:203–10. 10.1159/000531996 37611564

[87] Akiyama R Kawakami H Wong J Oishi I Nishinakamura R Kawakami Y. Sall4-Gli3 system in early limb progenitors is essential for the development of limb skeletal elements. Proc Natl Acad Sci U S A. (2015) 112:5075–80. 10.1073/pnas.1421949112 25848055 PMC4413345

[88] Watson JA Pantier R Jayachandran U Chhatbar K Alexander-Howden B Kruusvee V Structure of SALL4 zinc finger domain reveals link between AT-rich DNA binding and Okihiro syndrome. Life Sci Alliance. (2023) 6:e202201588. 10.26508/lsa.202201588 36635047 PMC9838217

[89] Wang W Yang N Wang L Zhu Y Chu X Xu W The TET-Sall4-BMP regulator*y* axis controls craniofacial cartilage development. Cell Rep. (2024) 43:113873. 10.1016/j.celrep.2024.113873 38427557

[90] Martin E Enriquez A Sparrow DB Humphreys DT McInerney-Leo AM Leo PJ Heterozygous loss of WBP11 function causes multiple congenital defects in humans and mice. Hum Mol Genet. (2020) 29:3662–78. 10.1093/hmg/ddaa258 33276377 PMC7823106

[91] Shin BK Kim J Kim MS Jang DH. Isolated congenital vertebral anomaly and Sprengel’s deformity in a WBP11 pathogenic variant. Eur J Med Genet. (2025) 75:105010. 10.1016/j.ejmg.2025.105010 40089178

[92] Moreno OM Sánchez AI Herreño A Giraldo G Suárez F Prieto JC Phenotypic characteristics and copy number variants in a cohort of Colombian patients with VACTERL association. Mol Syndromol. (2020) 11:271–83. 10.1159/000510910 33505230 PMC7802448

[93] Li Y Liu P Wang W Jia H Bai Y Yuan Z A novel genotype-phenotype between persistent-cloaca-related VACTERL and mutations of 8p23 and 12q23.1. Pediatr Res. (2024) 95:1246–53. 10.1038/s41390-023-02928-0 38135728

[94] Li M Zhang YL Zhang KL Li PP Lyu YH Liang YX Microdeletion on Xq27.1 in a Chinese VACTERL-like family with kidney and anal anomalies. Biomed Environ Sci. (2024) 37:503–10. 10.3967/bes2024.055 38843923

[95] van de Putte R van Rooij I Haanappel CP Marcelis CLM Brunner HG Addor MC Maternal risk factors for the VACTERL association: a EUROCAT case-control study. Birth Defects Res. (2020) 112:688–98. 10.1002/bdr2.1686 32319733 PMC7319423

[96] Solomon BD. VACTERL/VATER association. Orphanet J Rare Dis. (2011) 6:56. 10.1186/1750-1172-6-56 21846383 PMC3169446

[97] Keating ST El-Osta A. Epigenetics and metabolism. Circ Res. (2015) 116:715–36. 10.1161/circresaha.116.303936 25677519

[98] Krishnan V Jaganathan S Jayappa S Glasier C Choudhary A Ramakrishnaiah R Clinical and radiological evaluation of caudal regression syndrome. Pediatr Radiol. (2024) 54:1451–61. 10.1007/s00247-024-05945-1 38750326

[99] Stevens SJC Stumpel C Diderich KEM van Slegtenhorst MA Abbott MA Manning C The broader phenotypic spectrum of congenital caudal abnormalities associated with mutations in the caudal type homeobox 2 gene. Clin Genet. (2022) 101:183–9. 10.1111/cge.14076 34671974 PMC9153267

[100] Trimouille A Tingaud-Sequeira A Lacombe D Duelund Hjortshøj T Kreiborg S Buciek Hove H Description of a family with X-linked oculo-auriculo-vertebral spectrum associated with polyalanine tract expansion in ZIC3. Clin Genet. (2020) 98:384–9. 10.1111/cge.13811 32639022

[101] Thiem CE Stegmann JD Hilger AC Waffenschmidt L Bendixen C Köllges R Re-sequencing of candidate genes FOXF1, HSPA6, HAAO, and KYNU in 522 individuals with VATER/VACTERL, VACTER/VACTERL-like association, and isolated anorectal malformation. Birth Defects Res. (2022) 114:478–86. 10.1002/bdr2.2008 35362267

[102] Walters S Barkham B Bishop T Bernard J Coroyannakis C Thilaganathan B Fetal scoliosis: natural history and outcomes. J Am Acad Orthop Surg Glob Res Rev. (2024) 8:e24.00093. 10.5435/JAAOSGlobal-D-24-00093 38996079 PMC11132347

[103] Stack M Westmoreland T. Adolescent with VACTERL association presents with recurrent pneumonia. Cureus. (2020) 12:e10365. 10.7759/cureus.10365 33062488 PMC7550003

[104] Ćomić J Tilch E Riedhammer KM Brugger M Brunet T Eyring K Trio exome sequencing in VACTERL association. Kidney Int Rep. (2025) 10:877–91. 10.1016/j.ekir.2024.12.006 40225364 PMC11993224

[105] Dasgupta S Bhagwati NM Fatima A Sharma P Singh Kushwaha S Arora R Integration of prenatal sonography, fetal autopsy, histopathology and genetic tests in anomalous fetuses and diagnostic yield. J Obstet Gynaecol India. (2025) 75:180–9. 10.1007/s13224-025-02104-w 40390900 PMC12085723

[106] Groff E Cohen MC Steger F. The significance of clinical foetal autopsy for reproductive health care: an ethical analysis in the German context. Med Health Care Philos. (2025). 10.1007/s11019-025-10265-8 40178710 PMC12380977

[107] Bernardi P Graziadio C Rosa RF Pfeil JN Zen PR Paskulin GA. Fibular dimelia and mirror polydactyly of the foot in a girl presenting additional features of the VACTERL association. Sao Paulo Med J. (2010) 128:99–101. 10.1590/s1516-31802010000200011 20676578 PMC10938978

[108] Tamilselvan A Mohan MK. Long overlooked: adult VACTERL association unmasked by a large patent ductus arteriosus. Radiol Case Rep. (2025) 20:2758–62. 10.1016/j.radcr.2025.02.050 40165847 PMC11957573

[109] Oberg KC. Letter to the editor. Interesting, rare case, but VACTERL? J Neurosurg Case Lessons. (2024) 8:CASE24295. 10.3171/case24295 39186829 PMC11373700

[110] Harumatsu T Kaji T Nagano A Matsui M Murakami M Sugita K Successful thoracoscopic treatment for tracheoesophageal fistula and esophageal atresia of communicating bronchopulmonary foregut malformation group IB with dextrocardia: a case report of VACTERL association. Surg Case Rep. (2021) 7:11. 10.1186/s40792-020-01099-y 33409676 PMC7788125

[111] Yalcin S Bhatia AM He Z Wulkan ML. Short- and long-term outcomes of thoracoscopic and open repair for esophageal atresia and tracheoesophageal Fistula. J Pediatr Surg. (2024) 59:161662. 10.1016/j.jpedsurg.2024.08.002 39218728

[112] Al-Naimi A Hamad SG Zarroug A. Outcome of newborns with tracheoesophageal Fistula: an experience from a rapidly developing country: room for improvement. Pulm Med. (2022) 2022:6558309. 10.1155/2022/6558309 36507120 PMC9731754

[113] Masuko T Yanai T Toma M. Single-stage surgery for persistent cloaca with vertebral defects, anal atresia, cardiac defects, tracheoesophageal Fistula or atresia, renal anomalies, and limb defects (VACTERL) association: a case report on avoiding temporary colostomy. Cureus. (2025) 17:e82487. 10.7759/cureus.82487 40385907 PMC12085771

[114] Kassa AM Dahl M Strinnholm M Engstrand Lilja H. Attention difficulties and physical dysfunction common in children with complex congenital malformations: a study of preschool children with VACTERL association. Acta Paediatr. (2020) 109:783–9. 10.1111/apa.14566 30187514 PMC7154541

[115] Bergmann S Ritz LA Widenmann-Grolig A Jechalke S von Schweinitz D Hubertus J Swallowing-related quality of life in children with oesophageal atresia: a national cohort study. Eur J Pediatr. (2023) 182:275–83. 10.1007/s00431-022-04677-4 36331620 PMC9829586

[116] van Hoorn CE de Graaff JC Vlot J Wijnen RM Stolker RJ Schnater JM. Primary repair of esophageal atresia is followed by multiple diagnostic and surgical procedures. J Pediatr Surg. (2021) 56:2192–9. 10.1016/j.jpedsurg.2021.06.004 34229878

[117] Kassa AM Lilja HE. Neurodevelopmental outcomes in individuals with VACTERL association. A population-based cohort study. PLoS One. (2023) 18:e0288061. 10.1371/journal.pone.0288061 37384789 PMC10310046

[118] Ten Kate CA Rietman AB Kamphuis LS Gischler S Lee D Fruithof J Patient-driven healthcare recommendations for adults with esophageal atresia and their families. J Pediatr Surg. (2021) 56:1932–9. 10.1016/j.jpedsurg.2020.12.024 33455804

[119] Kassa AM Engvall G Dellenmark Blom M Engstrand Lilja H. Understanding of the transition to adult healthcare services among individuals with VACTERL association in Sweden: a qualitative study. PLoS One. (2022) 17:e0269163. 10.1371/journal.pone.0269163 35622841 PMC9140225

[120] Wang Z Sun Z Diao Y Wang Z Yang X Jiang B Identification of two novel SALL1 mutations in Chinese families with townes-brocks syndrome and literature review. Orphanet J Rare Dis. (2023) 18:250. 10.1186/s13023-023-02874-4 37644569 PMC10466882

[121] Graziano C Olivucci G. SALL1-related townes-brocks syndrome. In: Adam MP Feldman J Mirzaa GM Pagon RA Wallace SE Amemiya A, editors. GeneReviews(®) [Internet]. Seattle, WA: University of Washington, Seattle (2007). (updated 2024). 20301618

[122] Van Maldergem L Piard J Larizza L Wang LL. Baller-Gerold syndrome. In: Adam MP Feldman J Mirzaa GM Pagon RA Wallace SE Amemiya A, editors. GeneReviews(®) [Internet]. Seattle, WA: University of Washington, Seattle (2007). (updated 2018). 20301383

[123] Van Ravenswaaij-Arts CM Hefner M Blake K Martin DM. CHD7 disorder. In: Adam MP Feldman J Mirzaa GM Pagon RA Wallace SE Amemiya A, editors. GeneReviews(®) [Internet]. Seattle, WA: University of Washington, Seattle (2006). (updated 2022). 20301296

[124] Chen N Song S Bao X Zhu L. Update on Mayer-Rokitansky-Küster-Hauser syndrome. Front Med. (2022) 16:859–72. 10.1007/s11684-022-0969-3 36562950

[125] Herlin MK Petersen MB Brännström M. Mayer-Rokitansky-Küster-Hauser (MRKH) syndrome: a comprehensive update. Orphanet J Rare Dis. (2020) 15:214. 10.1186/s13023-020-01491-9 32819397 PMC7439721

[126] Schmitzer S Burcel M Dăscălescu D Popteanu IC. Goldenhar syndrome—ophthalmologist’s perspective. Rom J Ophthalmol. (2018) 62:96–104. 30206552 PMC6117527

[127] Bogusiak K Puch A Arkuszewski P. Goldenhar syndrome: current perspectives. World J Pediatr. (2017) 13:405–15. 10.1007/s12519-017-0048-z 28623555

[128] Shaik SP Arun Babu T. Goldenhar syndrome. BMJ Case Rep. (2024) 17:e259872. 10.1136/bcr-2024-259872 38839403

[129] Slavotinek AM McKusick-Kaufman syndrome. In: Adam MP Feldman J Mirzaa GM Pagon RA Wallace SE Amemiya A, editors. GeneReviews(®) [Internet]. Seattle, WA: University of Washington, Seattle (2002). (updated 2020). 20301675

[130] Baalaan KP Gurunathan N. Currarino triad. Pan Afr Med J. (2022) 41:143. 10.11604/pamj.2022.41.143.33419 35519164 PMC9046853

[131] Dworschak GC Reutter HM Ludwig M. Currarino syndrome: a comprehensive genetic review of a rare congenital disorder. Orphanet J Rare Dis. (2021) 16:167. 10.1186/s13023-021-01799-0 33836786 PMC8034116

[132] McDermott DA Fong JC Basson CT Holt-Oram syndrom”. In: Adam MP Feldman J Mirzaa GM Pagon RA Wallace SE Amemiya A, editors. GeneReviews(®) [Internet]. Seattle, WA: University of Washington, Seattle (2004). (updated 2019). 20301290

[133] Qudsieh H Aborajooh E Daradkeh A. Caudal regression syndrome: postnatal radiological diagnosis with literature review of 83 cases. Radiol Case Rep. (2022) 17:4636–41. 10.1016/j.radcr.2022.09.037 36204402 PMC9530488

[134] Kylat RI Bader M. Caudal regression syndrome. Children (Basel). (2020) 7:211. 10.3390/children7110211 33158301 PMC7694368

[135] Jasiewicz B Kacki W. Caudal regression Syndrome-A narrative review: an orthopedic point of view. Children (Basel). (2023) 10:589. 10.3390/children10030589 36980147 PMC10047641

[136] Mehta PA Ebens C Fanconi anemia. In: Adam MP Feldman J Mirzaa GM Pagon RA Wallace SE Amemiya A, editors. GeneReviews(®) [Internet]. Seattle, WA: University of Washington, Seattle (2002). (updated 2021). 20301575

